# Five-century record of climate and groundwater recharge variability in southern California

**DOI:** 10.1038/s41598-019-54560-w

**Published:** 2019-12-03

**Authors:** F. Manna, K. M. Walton, J. A. Cherry, B. L. Parker

**Affiliations:** G360 Institute for Groundwater Research, College of Engineering and Physical Sciences, University of Guelph, 50 Stone Road East, Guelph, ON N1G 2W1 Canada

**Keywords:** Hydrology, Hydrology

## Abstract

Modifications to the rates of water flowing from the surface to groundwater (groundwater recharge) due to climate variability are the most difficult to assess because of the lack of direct long-term observations. Here, we analyze the chloride salt distribution below the surface soil on a plateau near Los Angeles to reconstruct the amount of recharge that occurred in the last five centuries. Over this time interval, periods of major high and low recharge with different duration follow each other and this cyclicity is consistent with long-term atmospheric forcing patterns, such as the Pacific Decadal Oscillation. This study determines the range and the natural variability of recharge to groundwater, which sustains local freshwater flow system, and helps forecast future availability of groundwater resource in southern California, where water scarcity is critical to both local and global populations.

## Introduction

To gain insights about climate prior to the period of quantitative measurements, it is common to examine evidence from subsurface domains where it is “archived”^1^. The main sources of proxy data for paleoclimatic reconstructions can be grouped as follows: geologic (marine biogenic sediment and inorganic sediments), terrestrial (speleothems and glacial, periglacial, lacustrine, aeolian, pedological and shoreline deposits and features), glaciological (ice cores geochemistry, gas composition in air bubbles, microparticle concentration, physical properties), biological (tree rings, pollen, biota in lake sediments, corals, plant macrofossils), and historical (written records of environmental indicators)^1^. The temporal range of these natural archives varies from centuries to millennia, with a resolution from days (written historical records) to hundreds of years (marine sediments). Commonly, the information deduced from paleoclimatic studies pertain to air temperature, precipitation, atmospheric processes, and surface water but not groundwater. However, an important result of climate variability are modifications to the hydrologic cycle causing fluctuations in groundwater flow rates in sensitive regions, like discharge to streams and extraction via pumping to support agricultural and drinking water^2–4^. Groundwater provides nearly 50% of all drinking water worldwide, 66% of baseflow to surface water, and 43% of all water for irrigation in agriculture^5^. For this reason, estimating groundwater recharge and its temporal variability with emphasis on droughts is an important scientific endeavor with socioeconomic importance.

One approach for extracting information on the multidecadal variability of groundwater recharge over centuries involves the analysis of chloride (Cl) concentration in the porewater from core samples from the vadose zone (i.e., from above the water table)^6–8^. This method is based on the premise that rain percolates vertically downward through the vadose zone so that each year’s recharging water displaces downward the previous year’s water. Therefore, the pore water found at different depths in this zone represents the layering of successive inputs of rainfall from the most recent, just below land surface, to the oldest now situated at the water table. The age of the water upon arrival at the water table determines how far back in time the method encompasses. The Cl approach is applicable in recharge areas in semi-arid climate regions where there is no Cl released from the geologic media and no contributions from anthropogenic sources^9^. Where these geologic and anthropogenic conditions are met, the Cl in the vadose zone originates entirely from rainfall and dry atmospheric deposition on the land surface, which can be measured. After accounting for the Cl that is exported in surface runoff, the concentrating effect of surface evaporation and transpiration from the vegetation root zone causes the Cl concentration in the water that infiltrated below the root zone to be much greater than the equivalent aqueous concentration at the land surface. The concentration ratio of the Cl in the annual average atmospheric loading to the Cl in the pore water at any particular depth in the vadose zone is the recharge rate that produced the water found at that depth in the vadose zone. This is based on the premise that molecular diffusion has negligible effect on the Cl profile, in which case piston flow is a good approximation. The existing literature on the analysis of Cl concentration profiles as archives of past climate conditions pertains only to applications of the method in areas where sands and gravels (i.e., unconsolidated granular geologic deposits) comprise the vadose zone such that all infiltration occurs in intergranular porosity^10^. However, this study is the first application of the method to a fractured porous rock, where the potential role of the fractures on the flow in the unsaturated zone is addressed. Moreover, it is the first study to compare the results obtained from a fractured bedrock with past climatic conditions derived by analysis of tree-rings. The only other comparison between Cl profiles and tree-ring reconstruction was carried out in a sandy aquifer in the Chinese desert over 700 years^11^. Moreover, our investigation greatly extends the temporal scale of previous recharge studies in southern California that used observed water level fluctuations over a 70-year period^12,13^ and provides direct insights on the variability of the groundwater resources in a region that experiences severe drought conditions, impacting one of the continent’s most important agricultural production.

**Figure 1:**
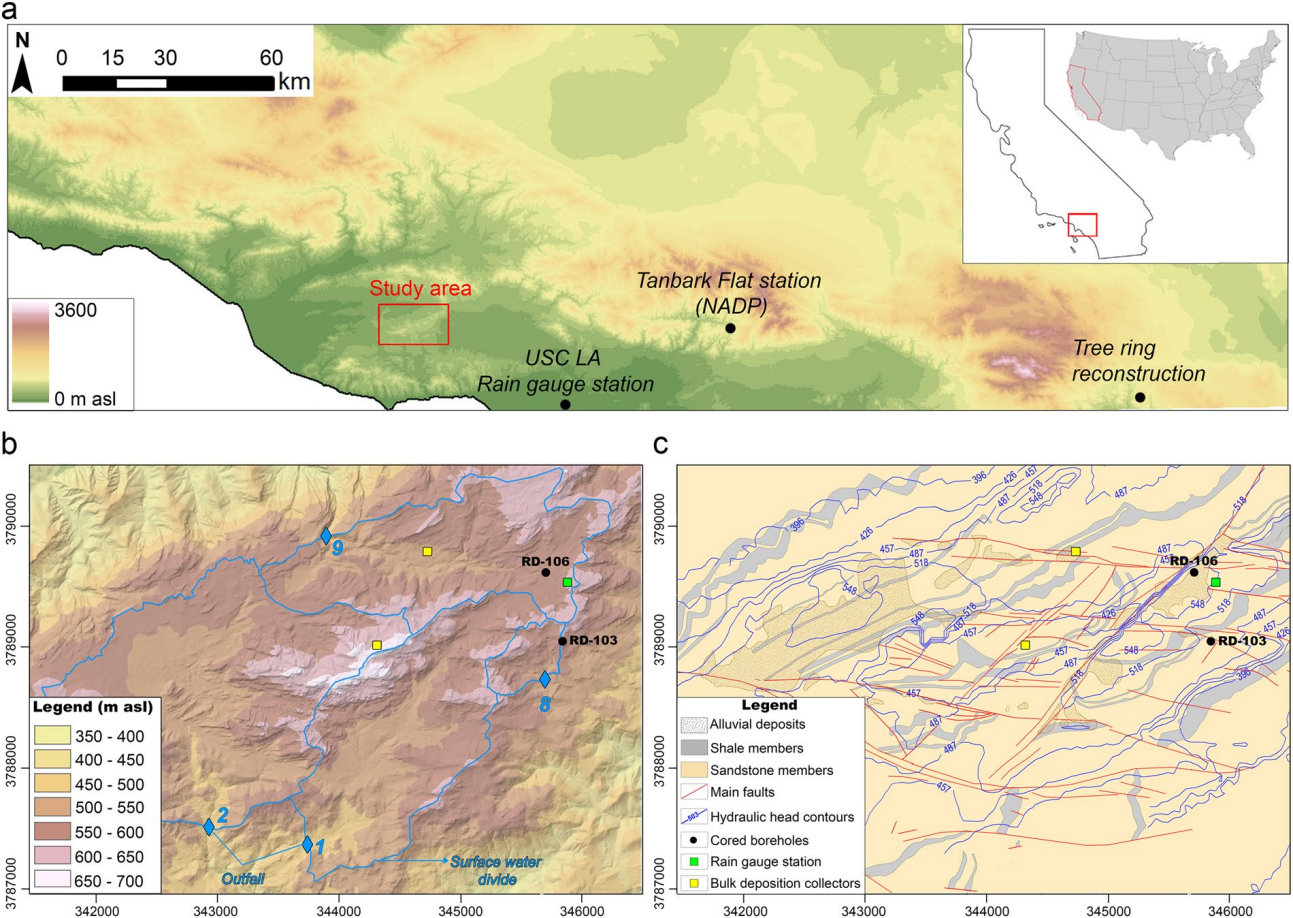
(**a**) Topographic map of southern California and location of the study area. (**b**) Topographic map of the study site with surface water divides and locations of the two cored holes, atmospheric deposition collectors and rain gauge station. (**c**) Hydrogeologic map of the site.

Specifically, we assess a vadose-zone porewater Cl profile to reconstruct the cycles of groundwater recharge variability with decadal resolution over nearly five centuries from a Cretaceous sandstone near Los Angeles, California. The study area was selected because groundwater in this region where is used for agricultural and drinking water and climate change concerns are mounting. Moreover, its climatic, hydrological and geological characteristics are well suited for insights on recharge going back centuries, long before rainfall and groundwater monitoring began in southern California. First, the site is located on an upland ridge, 45 km Northwest of Los Angeles and 25 km inland from the Pacific Ocean, standing about 300 m above the Simi and the San Fernando Valleys (Fig. 1a,b). Second, the majority (73%) of annual precipitation occurs during winter (December to March), whereas the summer season (June to August) is almost completely dry (10%). Conversely, the average monthly air temperature reaches a maximum in July and August (21.5 °C) and a minimum in January (10.4 °C). The mean annual potential evapotranspiration (1400 mm) exceeds the average precipitation (451 mm) creating semi-arid conditions^14,15^. Third, the vadose zone thickness is large (up to 80 meters), there is minimal overburden and the Cretaceous sandstone has relatively high matrix porosity (mean: 13.6%) and low matrix permeability (mean: 3 × 10^−9^ m s^−1^)^16^ (Fig. 1c). Therefore, although the rock is fractured, infiltration causing recharge occurs mostly by flow in the partially-water-saturated rock matrix blocks between fractures^17^. Fourth, there has been local monitoring of Cl in atmospheric dry fallout and rainfall and in the minimal streamflow volume measured at the outfalls (Fig. 1b,c). Fifth, the geologic Cl that existed when the sandstone formed has been removed by flushing during many millions of years of rainfall and groundwater flow^18^. Sixth, there is abundant information about the nature of the subsurface conditions based on studies of the vadose and groundwater zones that began in 1984, including studies of recharge^16,17,19,20^. About the latter, using the Cl mass balance method, Manna *et al*.^19^ estimated a site-wide, long-term average recharge of 19 mm, equal to 4.2% of the average annual precipitation. Later, from the analysis of eleven porewater profiles of Cl concentration obtained from high-resolution depth-discrete sampling of continuous cores was found that, on average, 80% of this total recharge occurs as flow in the matrix blocks in the vadose zone and that Cl concentration decreases and, therefore, recharge rates increase, as response to the removal of native vegetation^17^. Recently, a spatially-distributed hydrologic numerical model (MIKE SHE) representing surface water and groundwater for one catchment of the site, found that simulated recharge values span over three orders of magnitude as a function of topography, surface geology, and land use and that recharge occurs mainly at the end of the wet season and, occasionally, after exceptionally high-intensity precipitation events^20^.

## Approach to Reconstruct Recharge History

**Figure 2:**
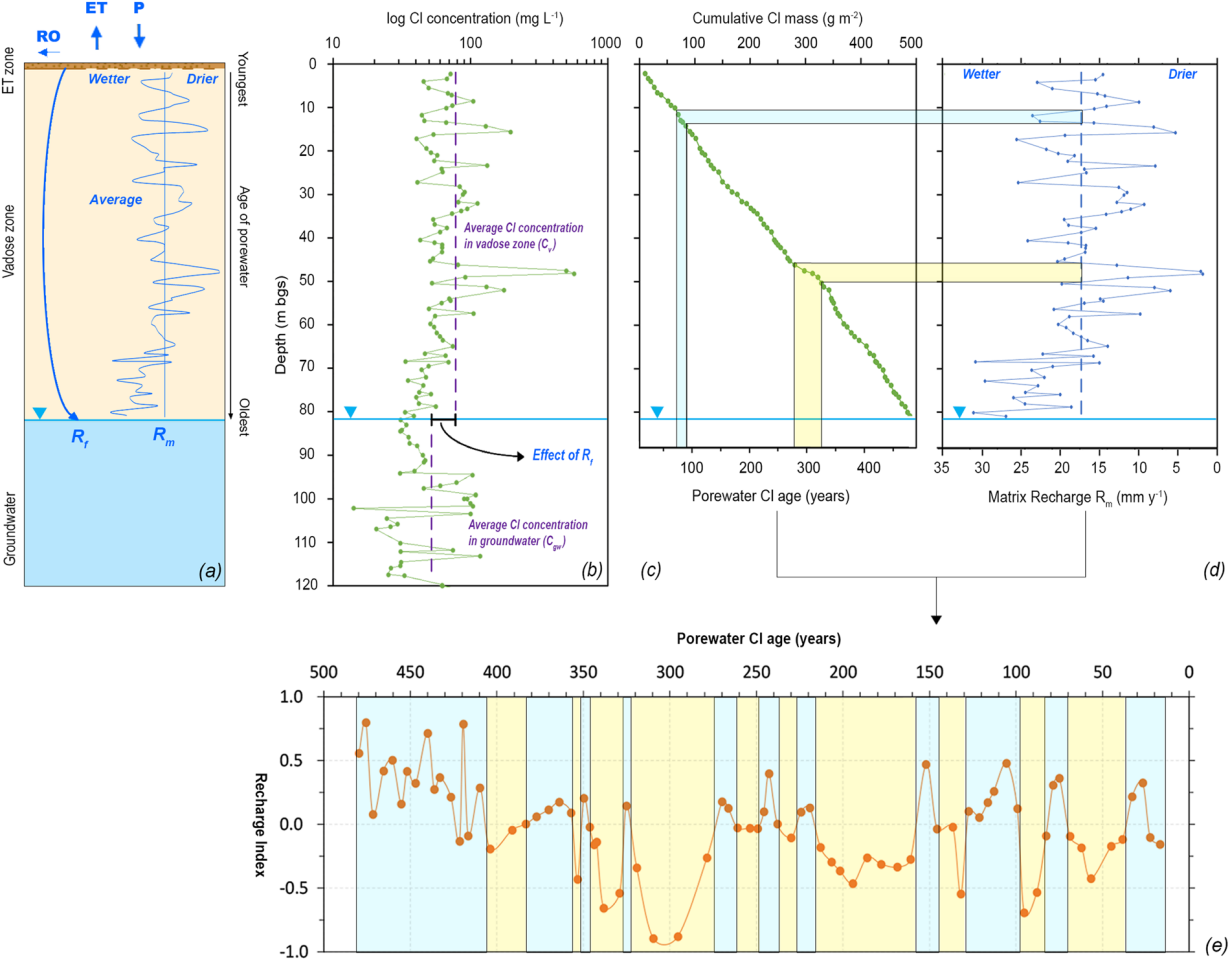
(**a**) Conceptual model of dual process recharge: *R*_*f*_ is the portion of total recharge that bypasses the matrix reaching directly the water table trough fractures; *R*_*m*_ is the recharge occurring through intergranular matrix flow with young recharge water displacing downward older water; (**b**) Cl concentration profile. Difference average concentration between vadose zone and groundwater is due to the presence of the lower-concentration fracture flow (*R*_*f*_). Cl concentration values in the vadose zone reflect variable temporal recharge conditions. (**c**) Cumulative Cl mass in vadose zone and Cl age of porewater. Age of porewater is calculated as the time needed to accumulate Cl at a given depth, assuming a constant atmospheric deposition rate, a piston-flow in the matrix and estimating mass of Cl in fracture flow. This allows to convert observations with depth to temporal observations. (**d**) Profile of matrix recharge (*R*_*m*_). Single Cl concentration values in the vadose zone represent recharge values for a specific period in the past. These intervals are calculated with Cl age (**c**). (**e**) Recharge Index representing temporal variability of recharge. For each point, porewater Cl age is estimated based on (**c**), recharge phases (wetter-blue and drier-yellow) are inferred from the deviation from the average of the depth-specific recharge values (**d**).

To investigate the vertical variability of Cl, we obtained two porewater profiles from high-resolution depth-discrete sampling of continuous cores at two locations, RD-103 and RD-106 (Fig. 1). To analyze the recharge history in this fractured sandstone, we adjusted the conventional approach used for granular aquifers by accounting for the possibility of a fast, preferential flow that reaches the water table directly via fractures bypassing the matrix (Fig. 2a). In granular aquifers, with no other process for enrichment below the root zone, the average concentration of Cl remains uniform between unsaturated and groundwater zone. Conversely, in fractured aquifers, the possible presence of a low-Cl-concentration flow, not enriched by the evapotranspiration, would cause Cl concentration in groundwater to be lower than that of the vadose zone. Also, water moving along the fracture network carries some Cl that should be removed from the input mass when calculating the Cl age of porewater in the vadose zone. Thus, in our recharge reconstruction, we firstly determined the contributions of the two components of the flow, matrix (*R*_*m*_) and fracture (*R*_*f*_), relative to the total recharge (Fig. 2a), based on the difference in average Cl concentrations in the vadose zone and in groundwater^21^ (Fig. 2b). Second, we focused on the vertical variations of Cl concentration in the vadose zone. We interpreted these as the effect of paleorecharge, with high Cl intervals associated with relatively dry periods and vice versa (Fig. 2b,d). The age of the porewater along each profile was determined with a new approach that integrates the Cl mass to a particular depth, then divides by the partitioned contribution of the rock-matrix component of Cl input flux, thus yielding a time to accumulate this mass (assuming a constant atmospheric Cl deposition rate) (Fig. 2c). Under the assumptions of piston-flow in the matrix, we relate the different Cl concentrations in the vadose zone to reconstruct a time series of past recharge conditions (Fig. 2e) (See supplementary information for details).

To check the consistency of our recharge reconstruction, we compared the trend of variability at RD-103, which has the thickest vadose zone (81 m), with the one observed at another location of the site (RD-106) and with the Pacific Decadal Oscillation (PDO) index time series, as reconstructed by tree-ring examination^22^. The PDO is a leading mode of multi-decadal variability in sea surface temperatures (SSTs) in the extratropical North Pacific^23^ and is defined by an index derived from the analysis of the SSTs. This index was calculated based on instrumental record from 1900 to the present^23^ and reconstructed back to 992 AD by MacDonald and Case^22^ analyzing trees located in southern California and Alberta. In southern California, positive phases of the PDO Index are associated with higher precipitation and vice versa^22^. In this region, past studies identified the influence of the PDO on precipitation^12,24^, streamflow^13^, lake levels^25^, wind^26^, and water levels in groundwater wells^12,13^.

### Precipitation variability

**Figure 3:**
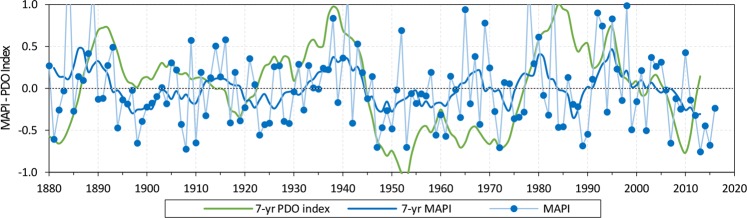
Mean Annual Precipitation Index (MAPI) and 7-year moving average of MAPI and PDO Index^22^ time series.

To verify the validity of using the PDO index as a proxy for past climate conditions at the site, PDO was compared to a Mean Annual Precipitation Index (MAPI), calculated using annual precipitation values from 1878 to 2016 (Fig. 3). The MAPI represents the deviation of the annual precipitation from the-long term average value. The MAPI time series was filtered with a moving average to determine the long-term trend, possible periodicity, and to minimize the effect of annual anomalies^27,28^. The MAPI varies from −0.76 (2013) to 1.69 (1884) around an average value, corresponding to the mean annual precipitation (450 mm). In the post-1875 period, positive index values occur 43% of the time. Five years (1884, 1889, 1941, 1978, 1983) have an index greater than 1, i.e., annual precipitation greater than 900 mm (Fig. 3). A cyclical pattern emerges from the 7-year moving average trend. There are six periods with values above the average (before 1892, 1912–1919, 1933–1944, 1964–1968, 1976–1983 and 1990–2003) and six with values below the average (1893–1911, 1920–1932, 1945–1963, 1969–1975, 1984–1989 and after 2004) (Fig. 3). These long-term fluctuations correspond to the 7-year moving average-smoothed PDO Index. The PDO Index for the same period shows three positive phases (1887–1915; 1923–1944; 1977–2005) and four negative phases (before 1887; 1916–1922; 1945–1976; after 2005) (Fig. 3). The consistency between the two-time series reconfirms the influence of the PDO fluctuation on the cyclical pattern of precipitation in southern California as observed by many others using different data, such as groundwater levels, lake levels and lacustrine deposits^12,13,25^. Differences may be attributed to additional climate forcing phenomena, such as the El Niño Southern Oscillation, affecting the trend of precipitation over shorter temporal scales^29^. As consequence, we use the PDO Index as proxy for precipitation for the long-term comparison between recharge and climatic indices.

### Recharge variability

**Figure 4:**
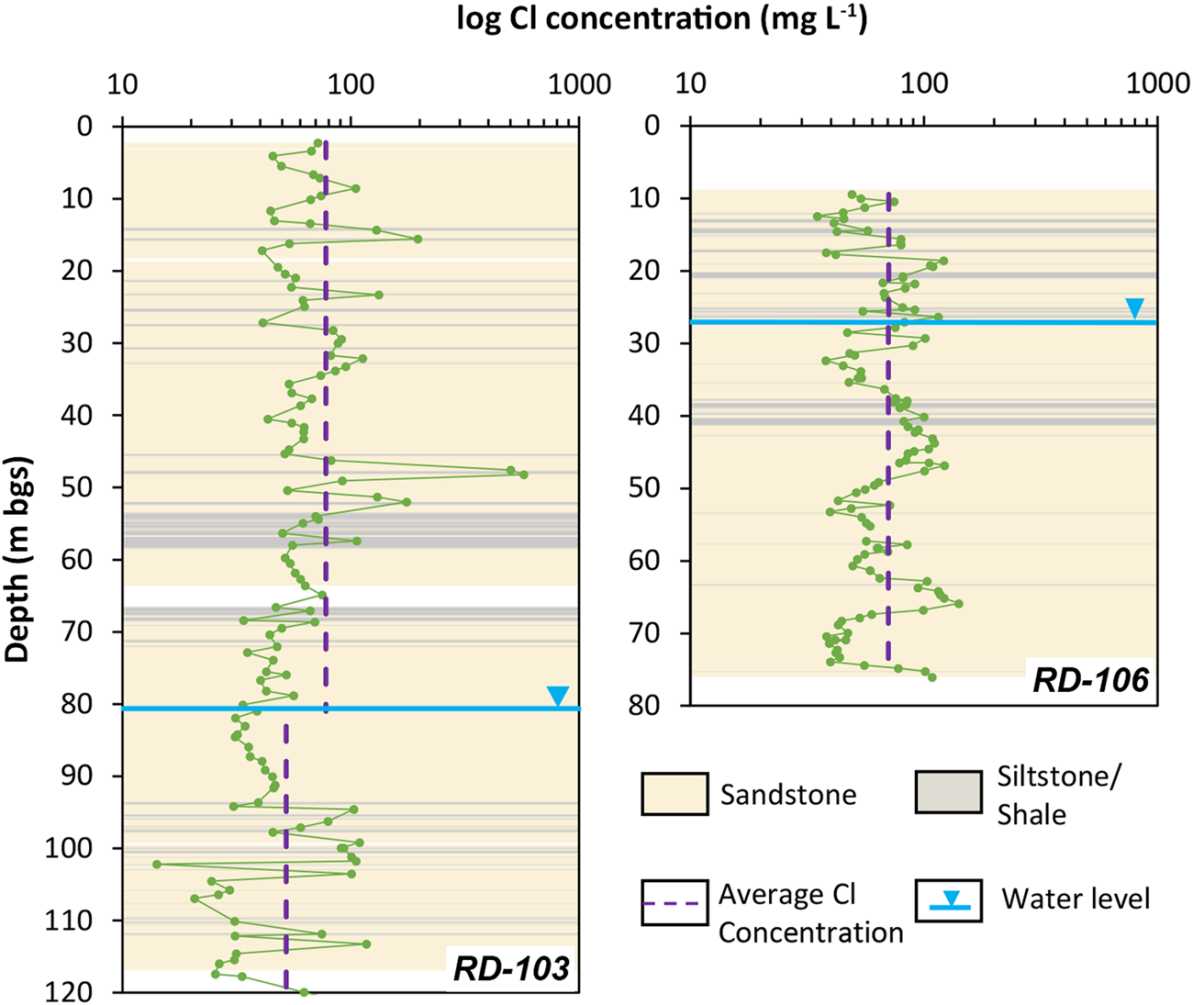
Cl profiles and stratigraphy at the two locations. Water table depth is 81 m at RD-103 and 27 m at RD-106.

**Figure 5:**
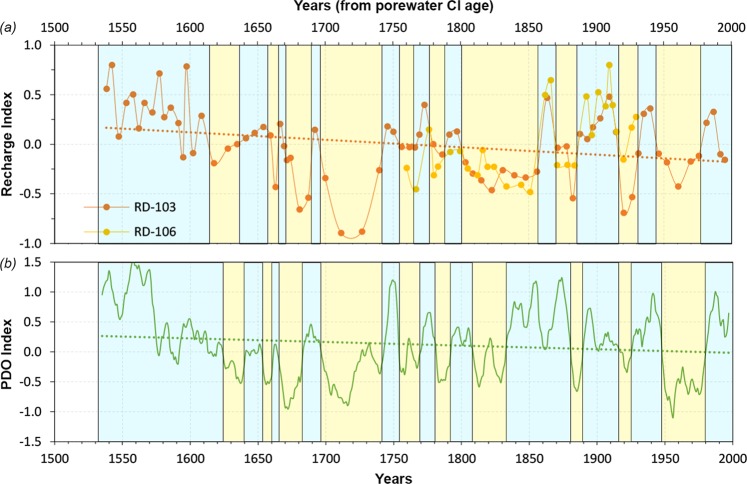
Comparison of recharge reconstructions with the PDO Index and their respective long-term trends. (**a**) Time series of Recharge Index calculated at RD-103 and RD-106 and linear trend for RD-103 only (longest time series); (**b**) 7-year moving average PDO Index time series and linear trend. Yellow and blue background colors represent drier and wetter than normal recharge or precipitation conditions respectively; dotted lines represent linear trends.

The average Cl concentrations in the vadose zone are 78.9 mg L^−1^ at RD-103 and 70.6 mg L^−1^ at RD-106, and in the groundwater zones are 51.6 mg L^−1^ at RD-103 and 71.1 mg L^−1^ at RD-106 (Fig. 4). Therefore, the average total recharge is 20 mm y^−1^ at RD-103 and 17 mm y^−1^ at RD-106, equal to 4.5% and 3.7% of the average annual precipitation. Given the same Cl concentration in the vadose zone and in groundwater at RD-106, we determine that recharge occurs entirely as matrix flow whereas at RD-103, 35% of the recharge occurs as flow through the fractures, which bypasses the matrix. Based on these recharge rates, the different thicknesses of the vadose zone, and the physical properties of the fractured porous rock at the two locations, we were able to reconstruct a 468-year history at RD-103 and 252-year history at RD-106 (Fig. 5a) (See supplementary information for details). Given the high frequency of the core sampling (1 m for RD-103 and 0.7 m for RD-106), each recharge value represents on average a 6-year period. To analyze the temporal variability of recharge at each location, we created a Recharge Index (RI), similar to the MAPI, representing the anomaly of each recharge value with respect to the mean value. RI values at RD-103 range between −0.89 (1705 to 1719) and +0.80 (1535 to 1539); the analysis of the linear trend shows a negative trend over the analyzed periods (dotted line: RI = −0.0007 × year + 1.32) (Fig. 5a). In addition to this trend, important oscillations with periods ranging from 5 to 77 years around the average values were observed. The longest positive phase, also with the highest recharge index values, was found between 1615 and 1538 (77 years) whereas prolonged dry periods were observed between 1698 and 1742 (44 years) and 1801 and 1855 (54 years). Range of variation at RD-106 varies between −0.48 (1861 to 1850) and +0.8 (1908 to 1911) and shows a nearly perfect overlap of peaks and troughs with RD-103. This coherent style of variability is the evidence of a similar change of conditions across the study area and reinforces the approach used at RD-103 to reconstruct recharge variations over a longer period (Fig. 5a).

To test our recharge reconstruction approach, we compared RD-103’s Recharge Index (RI) trend to the PDO Index (Fig. 5), reconstructed by MacDonald and Case analyzing tree rings for the last thousands of years (ftp://ftp.ncdc.noaa.gov/pub/data/paleo/treering/reconstructions/pdo-macdonald2005.txt). We compared the positive and negative phases (wetter and drier periods) of this index with the positive and negative phases of the recharge indices at the two locations. Principal positive and negative phases of the PDO Index are indicated in Fig. 5b. These changes of phase of the PDO Index are consistent with the fluctuations of the RI at RD-103, indicating impact of past climatic conditions on recharge history and, therefore, on the distribution of Cl in the vadose zone. Although not perfect, the similarity between the two patterns is remarkable, especially considering the distinct nature of the two datasets. Also, the linear trend of the PDO Index (PDOI) calculated over the same time interval of RD-103 shows a decreasing trend (dotted line: PDOI = −0.0007 × year + 1.38) consistent with that observed at RD-103, meaning a reduction of the recharge rate over the investigated time. The indices show substantially different behavior only between 1830 and 1855 when the PDO is positive and RIs are negative (Fig. 5). However, the fact that the RIs for both on-site core locations are negative supports a period of relatively drier conditions.

Considering only the last century, two main dry intervals were observed (1915 to 1931; 1945 to 1975) with recharge dropping to 70% lower than the average (Fig. 5a). These periods closely match those identified by analyzing the MAPI based on on-site precipitation (Fig. 3) and those observed by others based on precipitation data^30,31^, lake sediments^25^, and groundwater levels^32,33^. Given that the annual average precipitation for these previous recorded dry periods (381 and 394 mm y^−1^ respectively) is about 27% higher than that measured during the most recent drought (285 mm y^−1^ for the period 2011–2017), we should expect a stronger reduction in recharge to groundwater from this period. However, such a dry condition would not be new for the study area because recharge rates about 90% lower than average were found between 1710 and 1730. It is noteworthy that the ranges of variability of the RIs are larger than that of the MAPI (+0.48; −0.34); this is because of the non-linear relation between groundwater recharge and annual precipitation. Recharge in semi-arid areas, indeed, is not directly reliant on the total volume of precipitation but rather on the distribution throughout the year (seasonality)^20,34^ and intensities of the precipitation events^35,36^.

The consistency between the indices in Fig. 5 supports our recharge reconstruction approach and confirms the legitimacy of the use of Cl profiles as an archive of past climate conditions in fractured porous aquifers. This method is suitable for application in semi-arid regions where porous fractured sedimentary bedrock occurs in recharge areas and where the vadose zone is sufficiently thick and has moderately matrix permeability for retaining insights of past hydrologic conditions over the long term. Unlike other proxies, such as lacustrine sediments and tree-rings, the Cl profiles represent a more powerful tool because they show most directly the variability of the groundwater component of the hydrological cycle, which is generally the most difficult to assess because of the lack of direct observation.

At the study site, in southern California, we reconstructed the variability of recharge for the last 468 years, thus extending back by 4 centuries previous results obtained analyzing longest-term water level records^24,33^. The observed cyclical trend of groundwater recharge appears to be connected to the PDO index pattern and, therefore to atmospheric processes occurring at the global scale. Due to this relation, we propose that the PDO Index can be used as a qualitative indicator to forecast long-term recharge conditions. The uncovering of a recurrent oscillation with variable periodicity of groundwater recharge is fundamental to detect possible diverging trends caused by human-induced climate change. This observed multidecadal trend must also be considered by stakeholders and regulators when planning measures to avoid groundwater over-exploitation and to achieve sustainable management of water resources, so important to both local and global populations.

## Supplementary information

Supplementary Materials for ″Five-century record of climate and groundwater recharge variability in southern California″

## References

[1] Bradley, R. Paleoclimatology: Reconstructing Climates of the Quaternary. Third Edition edn, 696 (Elsevier, 2014).

[2] Crosbie RS (2013). Potential climate change effects on groundwater recharge in the High Plains Aquifer, USA. Water Resources Research.

[3] Lauffenburger ZH, Gurdak JJ, Hobza C, Woodward D, Wolf C (2018). Irrigated agriculture and future climate change effects on groundwater recharge, northern High Plains aquifer, USA. Agricultural Water Management.

[4] Meixner T (2016). Implications of projected climate change for groundwater recharge in the western United States. Journal of Hydrology.

[5] Smith, M., Cross, K., Paden, M. & Laben, P. Spring—managing groundwater sustainably. IUCN. (ISBN 978-2-8317-1789-0, 2016).

[6] Edmunds, W. & Walton, N. A geochemical and isotopic approach to recharge evaluation in semi-arid zones, past and present. *Arid-zone hydrology, investigation with isotope techniques. International Atomic Energy Agency, Vienna*, 47–68 (1980).

[7] Cook P, Edmunds W, Gaye C (1992). Estimating paleorecharge and paleoclimate from unsaturated zone profiles. Water Resources Research.

[8] Murphy EM, Ginn TR, Phillips JL (1996). Geochemical estimates of paleorecharge in the Pasco Basin: Evaluation of the chloride mass balance technique. Water Resources Research.

[9] Scanlon, B. R., Reedy, R. C. & Tachovsky, J. A. Semiarid unsaturated zone chloride profiles: Archives of past land use change impacts on water resources in the southern High Plains, United States. *Water Resources Research* **43** (2007).

[10] Stone A, Edmunds W (2016). Unsaturated zone hydrostratigraphies: a novel archive of past climates in dryland continental regions. Earth-science reviews.

[11] Gates JB, Edmunds WM, Ma J, Sheppard PR (2008). A 700-year history of groundwater recharge in the drylands of NW China. The Holocene.

[12] Hanson, R. T., Martin, P. & Koczot, K. M. Simulation of ground-water/surface-water flow in the Santa Clara-Calleguas ground-water basin, Ventura County, California (2003).

[13] Hanson RT, Dettinger MD (2005). Ground water/surface water responses to global climate simulations, Santa Clara-Calleguas Basin, Ventura, California. JAWRA. Journal of the American Water Resources Association.

[14] CIMIS. (Department of Land Arid and Water Resources, University of California, Davis and Water Efficiency Office, California Department of Water Resources, California Irrigation Management Unit. 1999).

[15] Hidalgo HG, Cayan DR, Dettinger MD (2005). Sources of variability of evapotranspiration in California. Journal of Hydrometeorology.

[16] Cherry, J. A., McWorther, D. B. & Parker, B. L. Site conceptual model for the migration and fate of contaminants in groundwater at the Santa Susana Field Laboratory, Simi, California (draft), vols 1–4. Association with the University of Guelph, Toronto, ON; MWH, Walnut Creek, CA (2009).

[17] Manna F, Walton KM, Cherry JA, Parker BL (2017). Mechanisms of recharge in a fractured porous rock aquifer in a semi-arid region. Journal of Hydrology.

[18] Pierce AA (2018). DFN-M field characterization of sandstone for a process-based site conceptual model and numerical simulations of TCE transport with degradation. Journal of contaminant hydrology.

[19] Manna F, Cherry JA, McWhorter DB, Parker BL (2016). Groundwater recharge assessment in an upland sandstone aquifer of southern California. Journal of Hydrology.

[20] Manna F (2019). Spatial and temporal variability of groundwater recharge in a sandstone aquifer in a semiarid region. Hydrology and Earth System Sciences.

[21] Sharma M, Hughes M (1985). Groundwater recharge estimation using chloride, deuterium and oxygen-18 profiles in the deep coastal sands of Western Australia. Journal of Hydrology.

[22] MacDonald, G. M. & Case, R. A. Variations in the Pacific Decadal Oscillation over the past millennium. *Geophysical Research Letters* **32** (2005).

[23] Mantua NJ, Hare SR, Zhang Y, Wallace JM, Francis RC (1997). A Pacific interdecadal climate oscillation with impacts on salmon production. Bulletin of the american Meteorological Society.

[24] Hanson R, Newhouse M, Dettinger M (2004). A methodology to asess relations between climatic variability and variations in hydrologic time series in the southwestern United States. Journal of Hydrology.

[25] Kirby M (2010). A Holocene record of Pacific decadal oscillation (PDO)-related hydrologic variability in southern California (Lake Elsinore, CA). Journal of Paleolimnology.

[26] Li AK, Paek H, Yu J-Y (2016). The changing influences of the AMO and PDO on the decadal variation of the Santa Ana winds. Environmental Research Letters.

[27] De Vita P, Allocca V, Manna F, Fabbrocino S (2012). Coupled decadal variability of the North Atlantic Oscillation, regional rainfall and karst spring discharges in the Campania region (southern Italy). Hydrology and Earth System Sciences.

[28] Manna F, Allocca V, Fusco F, Napolitano E, De Vita P (2013). Effect of the North Atlantic Oscillation on groundwater recharge in karst aquifers of the Cilento Geopark (Italy). Rendiconti Online Societa Geologica Italiana.

[29] Pavia EG, Graef F, Fuentes-Franco R (2016). Recent ENSO–PDO precipitation relationships in the Mediterranean California border region. Atmospheric Science Letters.

[30] Kunkel, K. E., Easterling, D. R., Redmond, K. & Hubbard, K. Temporal variations of extreme precipitation events in the United States: 1895–2000. *Geophysical research letters* **30** (2003).

[31] Woodhouse, C. A., Kunkel, K. E., Easterling, D. R. & Cook, E. R. The twentieth-century pluvial in the western United States. *Geophysical Research Letters* **32** (2005).

[32] Kuss AJM, Gurdak JJ (2014). Groundwater level response in US principal aquifers to ENSO, NAO, PDO, and AMO. Journal of Hydrology.

[33] Velasco EM, Gurdak JJ, Dickinson JE, Ferré T, Corona CR (2017). Interannual to multidecadal climate forcings on groundwater resources of the US West Coast. Journal of Hydrology: Regional Studies.

[34] Jasechko S (2014). The pronounced seasonality of global groundwater recharge. Water Resources Research.

[35] Taylor RG (2013). Evidence of the dependence of groundwater resources on extreme rainfall in East Africa. Nature Climate Change.

[36] Owor M, Taylor R, Tindimugaya C, Mwesigwa D (2009). Rainfall intensity and groundwater recharge: empirical evidence from the Upper Nile Basin. Environmental Research Letters.

