# Supplementary material for: Five-century record of climate and groundwater recharge variability in southern California
Source: Sci Rep. 2019 Dec 3;9:18215. doi: 10.1038/s41598-019-54560-w (PMC6890732; doi:10.1038/s41598-019-54560-w)
Supplement: Supplementary file 1 — Supplementary Materials for ″Five-century record of climate and groundwater recharge variability in southern California″ [file 41598_2019_54560_MOESM1_ESM.docx]

Supplementary Materials for

Five-century record of climate and groundwater recharge variability in southern California

F. Manna^1^, K. M. Walton^1^, J. A. Cherry^1^, B. L. Parker^1^

1. **Approach and methods**

**1.1 Reconstruction of recharge variability**

To reconstruct the trend of variability of groundwater recharge, we used the method presented in Manna et al. ^1^. It is an expansion over previous approaches used in granular aquifers ^2-5^ that considers the possibility of a fast, preferential flow that reaches the water table directly via fractures. This conceptual model of dual porosity recharge assumes that a portion of recharging water bypasses the matrix with negligible interaction. Although this represents a simplification of reality, we feel it is justified because of the dynamic and transient nature of fracture flow in the vadose zone and because the disparity in temporal scale of this method versus fast-flow transients (decades versus minutes/hours). At this stage, we also considered the chemical interaction between fractures and the matrix to be negligible because fractures in the vadose zone are usually dry and, therefore flow is primarily through the low permeability matrix. This very slow plug-flow generates a uniform front in the matrix reducing transverse dispersion or diffusion. The quantitation of the portion of recharge occurring as fracture flow is fundamental in the reconstruction of recharge variability time series. Chloride (Cl) age is defined as the time needed to accumulate the mass of Cl stored down to a certain depth in the vadose zone, calculated by assuming a constant Cl input. In the Cl-based method for granular aquifers, the input is represented from the average annual deposition rate (*q_a_* in mg m^-2^ yr^-1^) only, whereas the dual porosity approach requires partitioning the atmospheric rate into slow (*q_m_*) and fast (*q_f_*) components.

To quantify the contribution of the two components of flow, we used the approach developed by Sharma and Hughes ^6^ based on the difference in average Cl porewater concentration in the groundwater and in the vadose zone. In this dual flow regime, the total recharge is estimated as the ratio of *q_a_* to the Cl concentration in groundwater (*C_gw_*) as follows:

 (1).

The contributions of each flow component, matrix recharge (*R_m_*) and fracture recharge (*R_f_*), were calculated using the following equations:

 (2)

and

(3)

where *C_v_* is the average chloride concentration of porewater in the vadose zone and *C_a_* is the average bulk Cl concentration of water that infiltrates into the subsurface.

The time required to accumulate a given mass of Cl at depth *z* (*t_z_*) in the vadose zone (i.e., the age of the recharge water at depth *z*) may be calculated if both piston-flow and constant atmospheric inputs are assumed by the following:

 (4)

where *C’_vz_* is the mass of Cl per mass of dry rock (mg kg^-1^) and *ρ_b_* is the bulk density (kg m^-3^) in the *dz* interval (m), and *q_m_* (g m^-2^) is the portion of the atmospheric Cl deposition that infiltrates into the matrix.

However, the distribution of Cl in the vadose zone (and therefore the Cl age estimates) can be affected by diffusion processes. Fracture-matrix diffusive processes are excluded, as above, but vertical diffusive fluxes (*J* in (mg m^-2^ y^-1^)) in the porous medium are estimated by:

, (5)

where *D_e_* is the effective diffusion coefficient (0.0047 m^-2^ y^-1^ from Cherry et al. ^7^), *θ* is the volumetric water content, and *dC/dz* is the vertical concentration gradient. The resulting diffusive fluxes are very small in magnitude (from 0.1 to 0.4 mg m^-2^ y^-1^) relative to the total mass stored in the vadose zone (202.8 g m^-2^ for RD-106 and 490 g m^-2^ for RD-103). Therefore, we additionally exclude the effect of matrix diffusion in our calculations.

To determine *q_m_*, we assumed, as first approximation, that the Cl concentration in recharging water through fracture flow is equal to the concentration in the rainfall samples (*C_p_*). Consequently, the mass of Cl in fracture flow is:

 (6)

Therefore, the flux of Cl into the matrix is the difference between the total annual deposition rate and the portion of it that infiltrates into the subsurface as fracture flow:

 (7)

Given that RD-103 is located on the boundary of a catchment, we accounted for a removal of Cl as an effect of runoff by considering only 90% of the total *q_a_*. The same correction factor was not applied at RD-106 because runoff can both deliver or remove additional Cl at that location.

Once we established ages for porewater in the vadose zone, a time series of matrix recharge conditions was assembled. Each Cl measurement and its corresponding age yield a value for matrix recharge (*R_mz_*) for its interval:

 (8)

The form of Equation 8 is similar to what was proposed by Edmunds and Tyler ^3^ to identify positive and negative recharge phases. They reconstructed variability of recharge plotting Cl age versus the inverse of single values of Cl concentration in vadose zone samples.

**1.2 Site-specific recharge history reconstruction**

To determine the recharge history, we first determined the contributions of the two components of the flow, matrix (*R_m_*) and fracture (*R_f_*), relative to the total recharge. This analysis was based on the difference in average Cl concentrations in the vadose zone and in groundwater ^6^. The average Cl concentrations in the vadose zone are 78.9 mg L^-1^ at RD-103 and 70.6 mg L^-1^ at RD-106, and in the groundwater zones are 51.6 mg L^-1^ at RD-103 and 71.1 mg L^-1^ at RD-106 (Table 1). Therefore, the average total recharge is 20 mm y^-1^ at RD-103 and 17 mm y^-1^ at RD-106, equal to 4.5% and 3.7% of the average annual precipitation. Given the same Cl concentration in the vadose zone and in groundwater at RD-106, we determine that recharge occurs entirely as matrix flow whereas at RD-103, 35% of the recharge occurs as flow through the fractures, which bypasses the matrix (Table 1).

Second, we focused on the vertical variations of Cl concentration in the vadose zone. We interpreted these as the effect of paleorecharge, with high Cl intervals associated with relatively dry periods and vice versa. The age of the porewater along each profile was determined with a new approach that integrates the Cl mass to a particular depth, then divides by the partitioned contribution of the rock-matrix component of Cl input flux, thus yielding a time to accumulate this mass (assuming a constant atmospheric Cl deposition rate). Under the assumptions of piston-flow in the matrix, we relate the different Cl concentrations in the vadose zone to reconstruct a time series of past recharge conditions.

**1.3 Recharge Index (RI)**

Assuming that each discrete measurement of Cl concentration in the vadose zone is an expression of recharge conditions at a given time (Eq. 8), we created a recharge index (*RI*) to study the temporal variability of recharge as follows:

 (10)

where *R_mz_* is the matrix recharge from the corresponding *z*-interval, and is the average matrix recharge in the vadose zone.

**1.4 Mean Annual Precipitation Index (MAPI)**

We calculated a mean annual precipitation index (*MAPI*) to analyze the inter-annual variability of precipitation in the study area as follows ^8,9^:

(9)

where *P_i_* is the precipitation of year *i* and is the mean annual precipitation. The *MAPI* time series was filtered with a 7-year moving average to determine the long-term trend and possible periodicity. The time window was chosen based on the range of Cl ages in a single core sample interval (Eq. 4). Precipitation has been measured at the site continuously since 1960. The 57-year record is closely correlated (R^2^=0.90) with one measured for 139 years at the rain gauge station located about 50 km from the site, in downtown Los Angeles (<https://wrcc.dri.edu/cgi-bin/cliMAIN.pl?ca5115>). The existence of a good correlation justifies projecting the site-measured time series back to 1878 ^10^.

1. **Field data collection**

**2.1 Cl in atmospheric deposition**

Atmospheric Cl deposition rate was measured on site by two bulk deposition collectors (BDCs) designed to measure both dry and wet atmospheric deposition ^11^. Dry fallout deposited in a funnel that was then flushed into a collection bottle by precipitation events. The monitoring period was one year from March 2006 to March 2007. In that period, samples were collected three times: i) April 2006, ii) December 2006 and iii) March 2007. The total deposition rates for the three intervals are: i) 7.4, ii) 0.5, and iii) 1.3 mg m^-2^ d^-1^ for BDC-1 and i) 7.8, ii) 0.6, and iii) 1.7 mg m^-2^ d^-1^ for BDC-2. Therefore, the average deposition rate is 3.22 mg m^-2^ d^-1^ corresponding to an annual rate (*q_a_*) of 1.175 mg m^-2^.

To estimate the contributions of the two atmospheric deposition processes, nine rainfall samples representing wet deposition only, were collected and analyzed for Cl concentrations. Cl concentration ranged from 0.3 and 1.5 mg L^-1^ with an average of 0.7 mg L^-1^. This value is consistent with that measured for the period 1982-2016 at the Tanbark Flat station (0.49 mg L^-1^), which is the closest station of the National Atmospheric Deposition network located ~100 km east of the site. Given the average annual precipitation value for the site, the annual Cl deposition rate coming solely from precipitation is 315.7 mg m^-2^. This value represents 27% of the total atmospheric deposition. Therefore, dry fallout is the main atmospheric deposition process at the site, accounting for 73% of the total. This finding reinforces the assumption of a constant temporal atmospheric input through the analyzed period, which represents one of the main assumptions of the method. Dry deposition rate, indeed, depends mainly on the geographic location of the site and proximity to the ocean, whereas wet deposition varies with precipitation.

Considering a constant Cl concentration of 0.7 mg L^-1^ in rainfall and the standard deviation of precipitation of 212 mm y^-1^, then wet deposition ranges from 164 to 464 mg m^-2^ y^-1^. Combining this input’s range with a constant dry fallout rate of 859.6 mg m^-2^ y^-1^, the total annual deposition rate, *q_a_*, varies from 1026.9 to 1323.7 mg m^-2^ (±13%) ^1^.

**2.2 Cl in the vadose zone and groundwater**

Porewater samples were obtained from the two cored holes that were drilled in October 2011 (RD-103) and November 2012 (RD-106). Samples were collected from the top of the bedrock to the bottom of the borehole targeting both intact matrix blocks and close to observed fractures to detect any effect of matrix diffusion. The specific samples were selected based on field geological observations during drilling and coring operations. The resultant spatial resolution was 1 m for RD-103 (total of 118 samples, 78 in the vadose zone and 40 in groundwater) and 0.7 m for RD-106 (109 samples, 30 in the vadose zone and 79 in groundwater). Once collected, samples were immediately trimmed to remove the effect of drilling water, crushed using field apparatus, and preserved in vials ^12^. Each vial was weighed before sample preservation to calculate the mass of the wet, crushed rock; average field-condition sample mass was 35 g. In the laboratory, samples were then oven-dried at 105°C for 24 h to determine the gravimetric moisture content. Crushed rock was mixed with a known volume of deionized water to leach the salts for 48 h, with the leachate analyzed for Cl using inductively coupled plasma-mass spectrometry (ICP-MS; Agilent 8800). Finally, the porewater concentration was calculated using the sample-specific moisture content. For RD-103, where moisture content data were not available, the Cl concentration was calculated using average values of porosity and bulk density for more than 200 depth-discrete samples representing specific lithologies of the Chatsworth Formation from previous studies ^7,13,14^. This approximation results in an asymmetric error that ranges from -11 to +30% ^1^.

***Table 1****. CMB analysis and quantitation of the contribution of the two flow components to the total recharge.*

| **Core ID** | **Depth of first sample (m)** | **Groundwater depth (m)** | **Cl age (years)** | **Average Cl – VZ (mg L^-1^)** | **Average Cl – GW (mg L^-1^)** | **R**  **(mm)** | **R**  **(% P)** | **R_m_**  **(% R)** | **R_f_**  **(% R)** |
| --- | --- | --- | --- | --- | --- | --- | --- | --- | --- |
| RD-103 | 1.7 | 62.7 | 475 | 78.9 | 51.6 | 20 | 4.5 | 65 | 35 |
| RD-106 | 9.2 | 27.4 | 252 | 70.6 | 71.1 | 17 | 3.7 | 100 | 0 |

1 Manna, F., Walton, K. M., Cherry, J. A. & Parker, B. L. Mechanisms of recharge in a fractured porous rock aquifer in a semi-arid region. *Journal of Hydrology* **555**, 869-880, doi:10.1016/J.Jhydrol.2017.10.060 (2017).

2 Cook, P., Edmunds, W. & Gaye, C. Estimating paleorecharge and paleoclimate from unsaturated zone profiles. *Water Resources Research* **28**, 2721-2731 (1992).

3 Edmunds, W. & Tyler, S. Unsaturated zones as archives of past climates: toward a new proxy for continental regions. *Hydrogeology Journal* **10**, 216-228 (2002).

4 Edmunds, W. & Walton, N. A geochemical and isotopic approach to recharge evaluation in semi-arid zones, past and present. *Arid-zone hydrology, investigation with isotope techniques. International Atomic Energy Agency, Vienna*, 47-68 (1980).

5 Murphy, E. M., Ginn, T. R. & Phillips, J. L. Geochemical estimates of paleorecharge in the Pasco Basin: Evaluation of the chloride mass balance technique. *Water Resources Research* **32**, 2853-2868 (1996).

6 Sharma, M. & Hughes, M. Groundwater recharge estimation using chloride, deuterium and oxygen-18 profiles in the deep coastal sands of Western Australia. *Journal of Hydrology* **81**, 93-109 (1985).

7 Cherry, J. A., McWorther, D. B. & Parker, B. L. Site conceptual model for the migration and fate of contaminants in groundwater at the Santa Susana Field Laboratory, Simi, California (draft), vols 1–4. *Association with the University of Guelph, Toronto, ON; MWH, Walnut Creek, CA* (2009).

8 De Vita, P., Allocca, V., Manna, F. & Fabbrocino, S. Coupled decadal variability of the North Atlantic Oscillation, regional rainfall and karst spring discharges in the Campania region (southern Italy). *Hydrology and Earth System Sciences* **16**, 1389-1399, doi:10.5194/hess-16-1389-2012 (2012).

9 Manna, F., Allocca, V., Fusco, F., Napolitano, E. & De Vita, P. Effect of the North Atlantic Oscillation on groundwater recharge in karst aquifers of the Cilento Geopark (Italy). *Rendiconti Online Societa Geologica Italiana* **28**, 106-109 (2013).

10 Manna, F., Cherry, J. A., McWhorter, D. B. & Parker, B. L. Groundwater recharge assessment in an upland sandstone aquifer of southern California. *Journal of Hydrology* **541**, 787-799, doi:10.1016/j.jhydrol.2016.07.039 (2016).

11 MWH. Bulk chloride deposition collectors: installation and sampling procedures. Technical Memorandum. (2006).

12 Parker, B. L., Cherry, J. A. & Chapman, S. W. Discrete fracture network approach for studying contamination in fractured rock. *AQUA mundi* **3**, 101-116 (2012).

13 MWH. Draft-site wide groundwater remedial investigation report Santa Susana Field Laboratory, Ventura County, California. Prepared for The Boeing Company, NASA and U.S. DOE., (2009).

14 Amirtharaj, E., Ioannidis, M., Parker, B. & Tsakiroglou, C. Statistical synthesis of imaging and porosimetry data for the characterization of microstructure and transport properties of sandstones. *Transport in porous media* **86**, 135-154 (2011).
